# Altered *in vitro* muscle differentiation in X-linked myopathy with excessive autophagy

**DOI:** 10.1242/dmm.041244

**Published:** 2020-01-10

**Authors:** Stephanie A. Fernandes, Camila F. Almeida, Lucas S. Souza, Monize Lazar, Paula Onofre-Oliveira, Guilherme L. Yamamoto, Letícia Nogueira, Letícia Y. Tasaki, Rafaela R. Cardoso, Rita C. M. Pavanello, Helga C. A. Silva, Merari F. R. Ferrari, Anne Bigot, Vincent Mouly, Mariz Vainzof

**Affiliations:** 1 Human Genome and Stem-Cell Research Center, Biosciences Institute, University of São Paulo, 05508-900 São Paulo, Brazil; 2 Department of Neurology and Neurosurgery, Division of Neuromuscular Disorders, Federal University of São Paulo, 04023-062 São Paulo, Brazil; 3 Sorbonne Université, Inserm, Institut de Myologie, U974, Center for Research in Myology, 47 Boulevard de l'hôpital, 75013 Paris, France

**Keywords:** XMEA, Autophagy, Muscle, Myogenesis

## Abstract

X-linked myopathy with excessive autophagy (XMEA) is a genetic disease associated with weakness of the proximal muscles. It is caused by mutations in the *VMA21* gene, coding for a chaperone that functions in the vacuolar ATPase (v-ATPase) assembly. Mutations associated with lower content of assembled v-ATPases lead to an increase in lysosomal pH, culminating in partial blockage of macroautophagy, with accumulation of vacuoles of undigested content. Here, we studied a 5-year-old boy affected by XMEA, caused by a small indel in the *VMA21* gene. Detection of sarcoplasmic Lc3 (also known as MAP1LC3B)-positive vacuoles in his muscle biopsy confirmed an autophagy defect. To understand how autophagy is regulated in XMEA myogenesis, we used patient-derived muscle cells to evaluate autophagy during *in vitro* muscle differentiation. An increase in lysosomal pH was observed in the patient's cells, compatible with predicted functional defect of his mutation. Additionally, there was an increase in autophagic flux in XMEA myotubes. Interestingly, we observed that differentiation of XMEA myoblasts was altered, with increased myotube formation observed through a higher fusion index, which was not dependent on lysosomal acidification. Moreover, no variation in the expression of myogenic factors nor the presence of regenerating fibers in the patient's muscle were observed. Myoblast fusion is a tightly regulated process; therefore, the uncontrolled fusion of XMEA myoblasts might generate cells that are not as functional as normal muscle cells. Our data provide new evidence on the reason for predominant muscle involvement in the context of the XMEA phenotype.

This article has an associated First Person interview with the first author of the paper.

## Abstract (precis)

**Summary:** Here, we show that in X-linked myopathy with excessive autophagy there is increased fusion of myoblasts, which is not caused by the primary lysosomal acidification defect.

## INTRODUCTION

X-linked myopathy with excessive autophagy (XMEA, OMIM 310440) is a muscle disorder with a commonly childhood onset that affects only males. XMEA patients have weakness in the proximal muscles of lower extremities that progresses to other muscles until loss of ambulation occurs at around 50 years of age (Kalimo et al., 1988; Chabrol et al., 2001). Late-onset forms of the disease have also been described (Crockett et al., 2014). Although cardiac involvement is not a classic feature (Kalimo et al., 1988; Saraste et al., 2015), it has recently been reported that vacuolation of cardiac muscle can also be a feature in more severe cases (Munteanu et al., 2017). In muscle biopsies, membrane-bound sarcoplasmic vacuoles can be observed (Kalimo et al., 1988). Only a few XMEA families have been identified worldwide. This myopathy was initially described in a large Finnish family, with five affected members and a clear X-linked recessive inheritance. The clinical course was mild, and the patients were affected by a slowly progressive muscle weakness mainly in the legs but did not lose their ability to walk. There was no evidence of cardiac or neural involvement and serum creatine kinase was elevated. By electron microscopy, an excessive number of autophagic vacuoles with staining properties of lysosomes was observed (Saviranta et al., 1988; Kalimo et al., 1988). Additional sporadic families with similar phenotypes were described afterwards by different groups (Villanova et al., 1995; Minassian et al., 2002). All patients were males with childhood-onset progressive weakness and wasting of skeletal muscle. Proximal muscles of the lower extremities were always initially and later predominantly affected and no other organ system was affected clinically. Later, Yan et al. (2005) reported the first Chinese-American family with XMEA, but the two affected male siblings had a severe congenital form of the disease.

XMEA is a disease caused by hypomorphic alleles of the vacuolar ATPase assembly factor (*VMA21*) gene (Ramachandran et al., 2013). The protein encoded by this gene is a chaperone that is involved in the correct assembly of the vacuolar ATPase (v-ATPase), which is the proton pump involved in lysosomal acidification. In XMEA, there is a decrease in assembled v-ATPases, leading to a slightly increased lysosomal pH, which in turn culminates in partial blockage of the degradative step of macroautophagy. As a consequence, a feedback mechanism is activated with higher levels of macroautophagic induction, leading to accumulation of vesicles with undigested content inside muscle fibers (Ramachandran et al., 2013). Six different single-nucleotide substitutions in the *VMA21* gene and two non-coding microdeletions were identified in 14 families with XMEA (Ramachandran et al., 2013). Four were intronic, and, in two of them, the IVS1-27A base branch point was involved. These mutations result in a 32-58% reduction in *VMA21* mRNA.

Macroautophagy, hereafter referred to as autophagy, is a recycling process for proteins and damaged organelles via lysosomes. It occurs through the formation of double-membraned structures called autophagosomes, which engulf the cargo and fuse with the lysosome for degradation. This pathway has been increasingly described as essential for muscle function and structure (Masiero et al., 2009; Mammucari et al., 2007; Zhao et al., 2007). In previous years, autophagy has also been strongly implicated in differentiation of progenitor muscle cells (myoblasts) into myotubes, which are the cells that undergo maturation to form adult muscle fibers. Studies investigating the differentiation of immortalized mouse myoblasts (C2C12 cells) showed that autophagy is increased during myotube formation. This increase is essential to protect those cells against apoptosis-mediated cell death (McMillan and Quadrilatero, 2014). Later, the autophagy pathway was implicated in the mitochondrial degradation that needs to occur in myoblasts to allow posterior mitochondrial biogenesis for the appropriate metabolism of myotubes (Sin et al., 2016). Those results were corroborated by studies with satellite cells that are muscle stem cells, in which autophagy plays an essential role in myotube formation (Fortini et al., 2016).

Here, we describe the first XMEA Brazilian family, with a small indel in the *VMA21* gene, and we investigated how autophagy is regulated in XMEA muscle progenitor cells. We found less-acidic lysosomes, an increase in autophagic flux in XMEA myotubes and increased myotube formation, with a higher fusion index. However, no variation in myogenic factors and no regeneration within the biopsy was found. Our findings address new pathomechanisms of this rare disease.

## RESULTS

### The clinical description and family history of the proband are compatible with XMEA

**Fig. 1.**
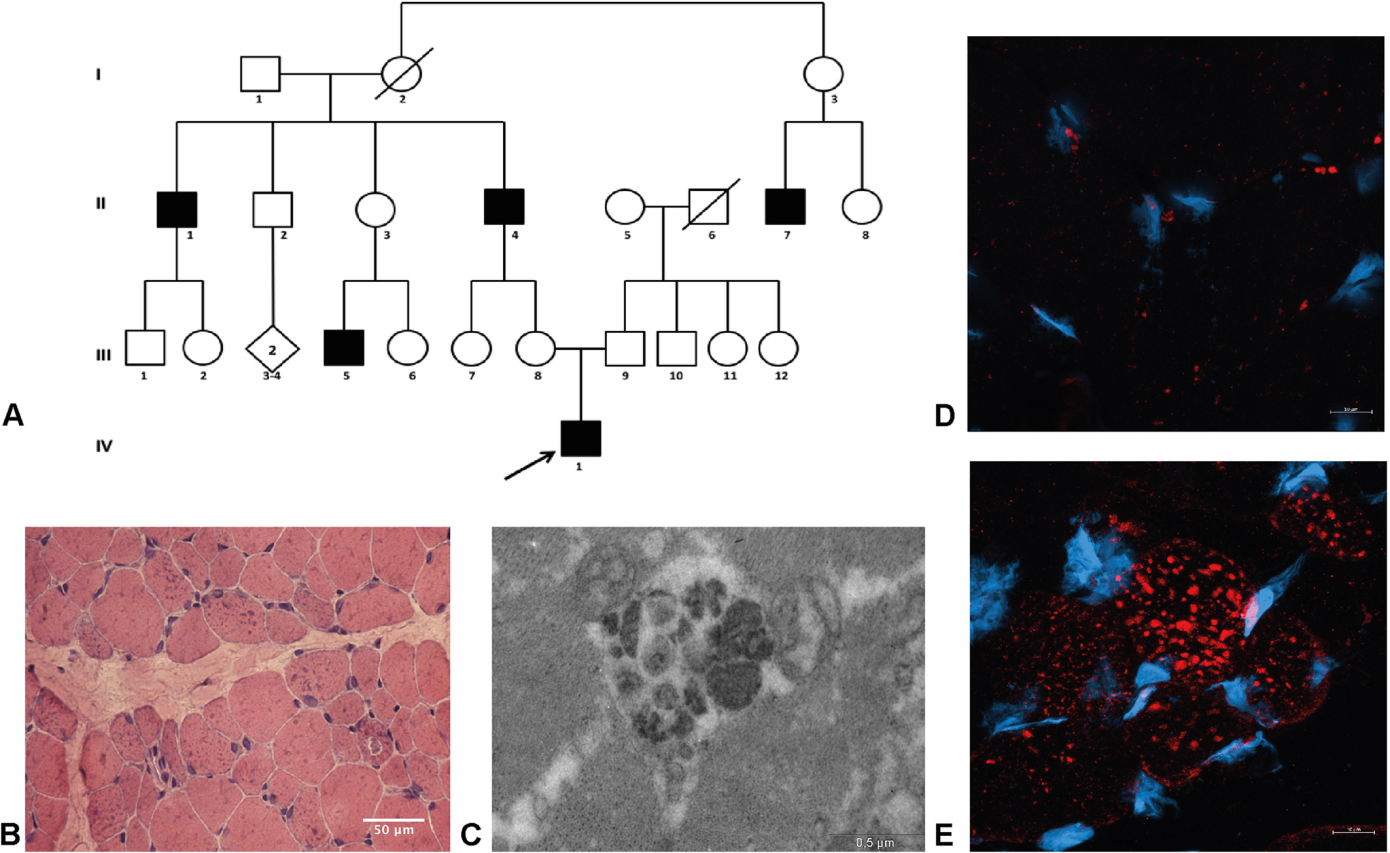
**The XMEA patient has an accumulation of autophagic vacuoles in his muscle.** (A) Pedigree of the patient's family. (B) H&E staining showing the presence of basophilic inclusions. Magnification 200×, light microscopy. Scale bar: 50 μm. (C) Electron micrograph of a vacuole inside a muscle fiber. Scale bar: 0.5 µm. (D,E) Lc3 immunostaining in a muscle biopsy of a control (D) and XMEA patient (E), with a remarkable accumulation of Lc3-positive vacuoles in the patient muscle. Red, Lc3; blue, DAPI, Magnification 630×, confocal microscopy. Scale bars: 10 µm.

The 5-year-old propositus presented a characteristic dystrophic phenotype. He was born by cesarean delivery, in the eighth month of pregnancy, due to maternal hypertension. He showed normal mental development, walked at the age of 2 years, with weight and height always below normal. The boy could walk normally and on his heels and jump a little, but needed support from the hands to lift off the ground. Subsequently, he showed difficulties with running, climbing stairs and raising from the floor. He complained of pain in the calves, but no calf hypertrophy or joint contractures were observed. His creatine kinase level was 1330 U/l (normal value=195 U/l) and an electrocardiogram showed incomplete right bundle branch conduction. Family history revealed a clear X-linked recessive pattern of inheritance, with five affected males linked through asymptomatic females. The affected maternal grandfather, aged 48 years, was wheelchair bound from the age of 30, presenting also cardiac alterations and joint contractures in the upper limbs. He began to walk on tiptoes at the age of 25, and could not raise his arms by the age of 48. Additional affected members of this family include a brother, a nephew and a cousin of the grandfather (Fig. 1A).

### Vacuolation and Lc3-positive puncta are prominent in the muscle biopsy

Histopathological analysis of muscle biopsy sections stained with Hematoxylin-Eosin (H&E) showed a notable presence of vacuoles in muscle fibers, as observed by the basophilic inclusions in Fig. 1B. It was also possible to observe variation in fiber caliber. Analysis of muscle proteins by immunofluorescence revealed the presence of dystrophin, α2-laminin, and α-, β- and γ-sarcoglycan in the sarcolemma (data not shown). Membrane α2-laminin staining was also detected inside the autophagic vacuoles. In addition, emerin detection revealed the presence of the protein in the nucleus.

Electron microscopy showed the presence of large autophagic vacuoles inside muscle fibers, with double membranes (Fig. 1C). Those vacuoles were confirmed as being autophagic by immunostaining for Lc3 (also known as MAP1LC3B) protein, which is present in the membranes of autophagosomes. In this case, accumulation of Lc3-positive vacuoles was observed in the XMEA muscle biopsy but not in the control biopsy (Fig. 1D,E).

### The XMEA patient has a mutation in the *VMA21* gene and reduced expression of *VMA21* mRNA

**Fig. 2.**
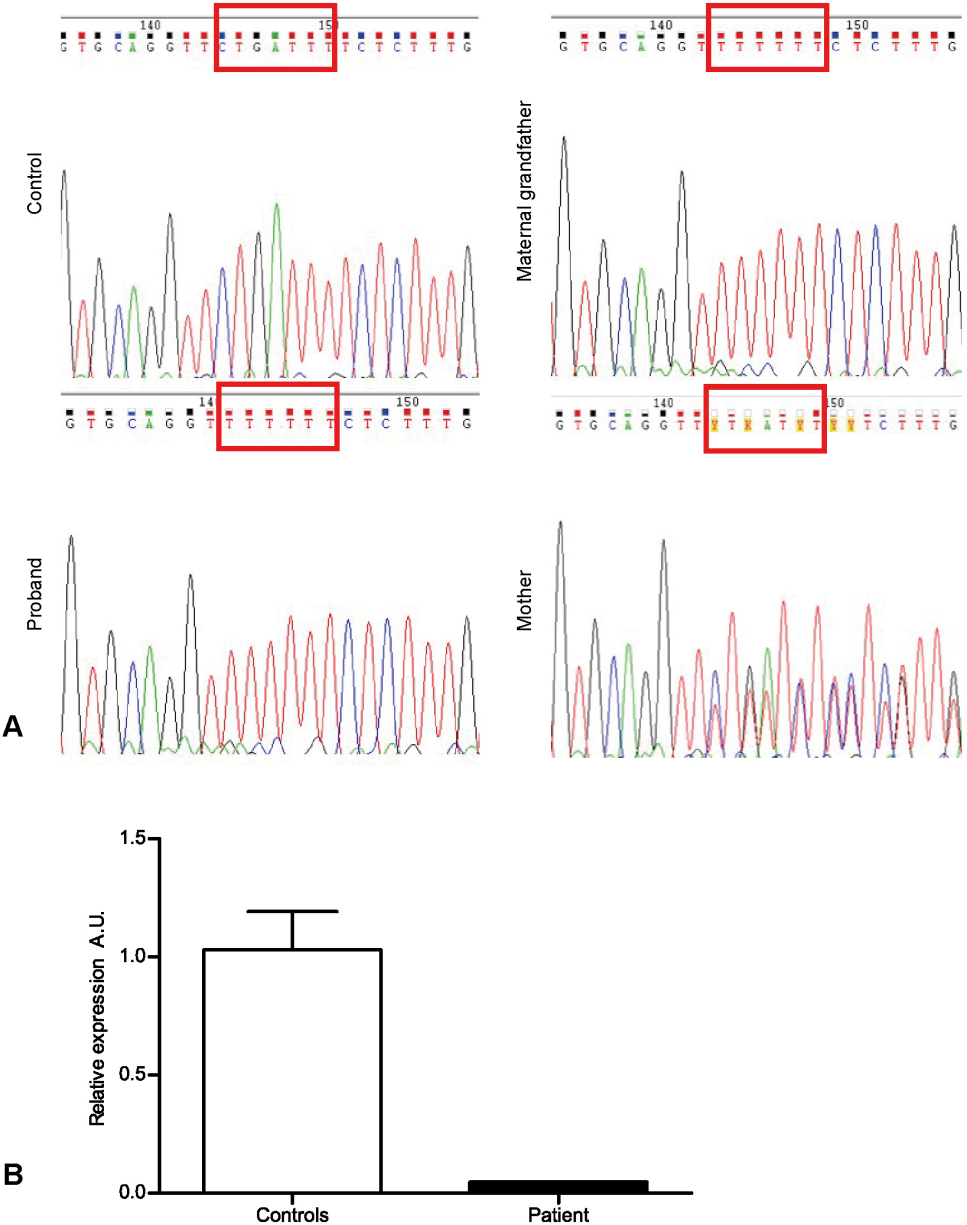
**The diagnosis of XMEA is confirmed in the first-described Brazilian family.** (A) A microdeletion was observed in the *VMA21* gene in the patient, his mother and his maternal grandfather. (B) *VMA21* mRNA expression was reduced in the patient compared to three male controls. A.U., arbitrary units.

Whole-exome sequencing was undertaken for the index case on an Illumina HiSeq 2000 platform, and a first screening for rare variants failed to identify any candidate mutations in this family, excluding mutations in known genes associated with his phenotype. As recent findings revealed that *VMA21* was a strong candidate gene to be causative of XMEA, a detailed evaluation of this gene was carried out, including the flanking intronic regions. We identified a hemizygous small deletion in intron 1 of the *VMA21* gene (NM_001017980.3: c.54-30_54-27delinsT), including the IVS1-27A base previously described. The presence of the mutation in the patient, his grandfather and his heterozygous mother was confirmed through Sanger sequencing (Fig. 2A). Analysis of *VMA21* mRNA expression in the patient's muscle biopsy showed a clear reduction in the expression of this gene in the muscle, compared to the muscle of three male controls (Fig. 2B).

### Progenitor muscle cells with mutation in the *VMA21* gene preserve their proliferation and differentiation capacities

As the patient has the characteristic pathology of XMEA, we isolated progenitor muscle cells from his muscle biopsy. Myoblasts were successfully obtained, and a cellular model of the disease was generated by immortalization of those cells (Mamchaoui et al., 2011). First, we confirmed that the cells are representative of the pathology by studying *VMA21* gene expression, which showed that expression is reduced in all studied time points in the patient-derived cells but not in the control cells (Fig. S1A). Interestingly, we observed a trend for higher *VMA21* expression with differentiation after 3 and 6 days in control cells, which might indicate a novel crosstalk between myogenesis and v-ATPase assembly. The immortalization was proven to be successful, and the cells retained their ability to proliferate and to form myotubes (Fig. S1B).

### XMEA myoblasts and myotubes have fewer acidic lysosomes

**Fig. 3.**
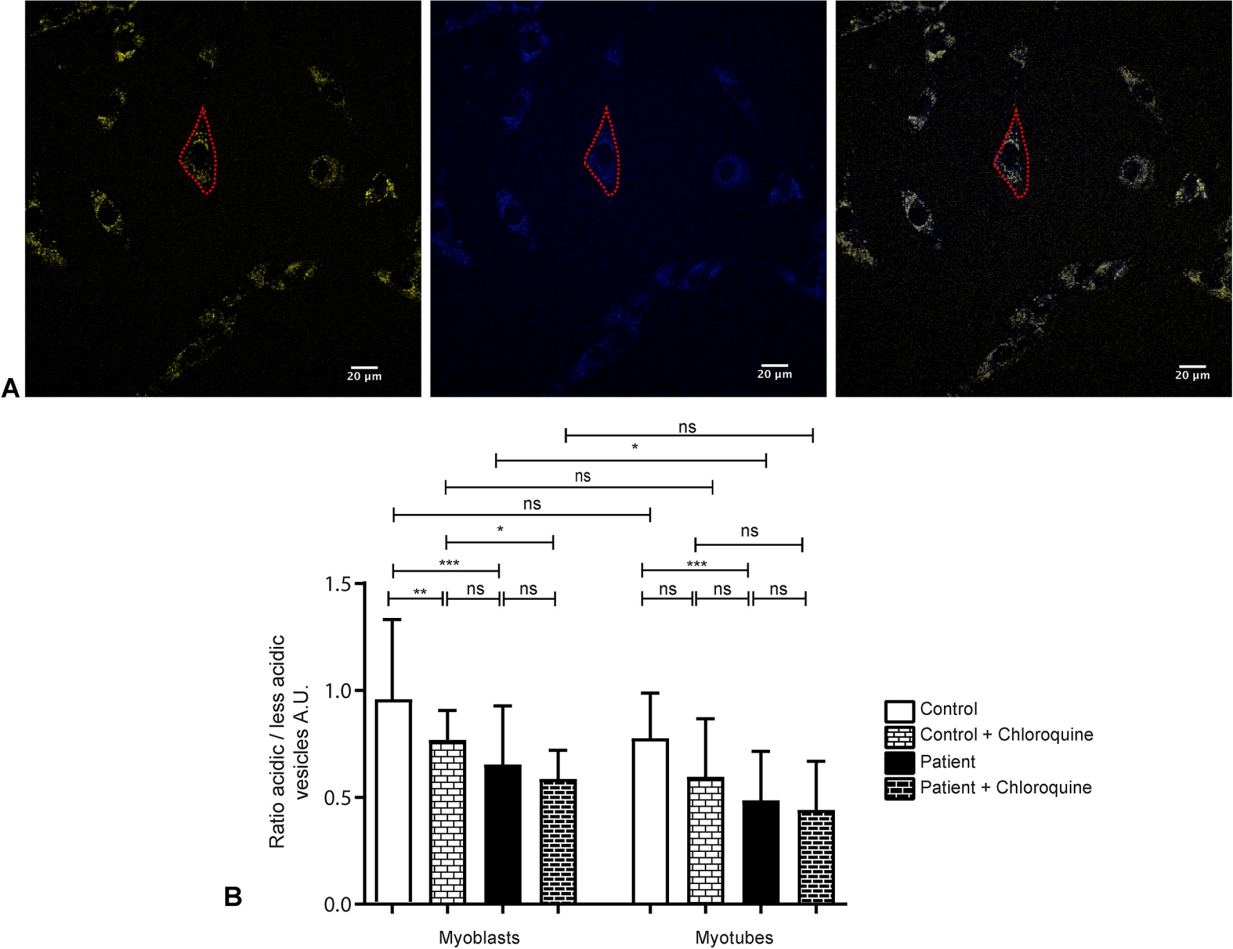
**XMEA cells are less acidic** **than control** **cells.** (A) The Lysosensor probe has yellow fluorescence in acidic organelles, in contrast to the blue fluorescence in more basic compartments. The ratio between those is used to estimate the number of acidic organelles within the area of myoblasts or myotubes. Red dashed lines show the delimitation of a single myoblast. Scale bars: 20 μm. (B) The ratio between acidic and less-acidic vesicles showed that myoblasts and myotubes from the XMEA patient have fewer acidic vesicles than control cells. Chloroquine treatment in control myoblasts mimicked the XMEA phenotype, but not in myotubes. We measured 95 cells for control myoblasts, 49 cells for control myoblasts treated with chloroquine, 87 cells for patient myoblasts, 29 cells for patient myoblasts treated with chloroquine, 27 cells for control myotubes, 15 cells for control myotubes treated with chloroquine, 30 cells for patient myotubes and 23 cells for patient myotubes treated with chloroquine. Two independent experiments were performed for untreated cells and one for chloroquine-treated cells. Experiments were performed in serum-free medium. Data are presented as mean±s.d. *P<0.05, **P<0.01, ***P<0.001; ns, non-significant. Three-way ANOVA with Bonferroni post hoc for multiple comparisons. A.U., arbitrary units.

The primary defect of XMEA is causative of an increased lysosomal pH. To investigate whether XMEA cells have the expected phenotype, we performed Lysosensor analysis. The Lysosensor probe has blue fluorescence in neutral environments, and in acidic organelles, such as lysosomes, it turns yellow. We then calculated the ratio between yellow and blue vesicles within the area of each myoblast or myotube, as an indication of lysosomal acidification (Fig. 3A). Ratio analysis showed that in control and patient cells there is no difference in acidification between myoblasts and myotubes (Fig. 3B). XMEA myoblasts and myotubes, however, accumulate fewer acidic vesicles than control cells (Fig. 3B), demonstrating that these cells have the expected phenotype of XMEA pathology. In addition, it suggests the initial step of defective autophagy in patient-derived myoblasts and myotubes. Moreover, another independent probe that relies on lysosomal acidification, namely Lysotracker, also showed that myoblasts from the patient have lower acidification (Fig. S2B). As a control, Mitotracker was used and no changes were observed regarding mitochondrial content (Fig. S2A).

To confirm that the reduced ratio between acidic/less-acidic vesicles observed in XMEA cells is indeed due to a decrease in lysosomal acidification, we treated both control and patient-derived cells with chloroquine. Chloroquine treatment induces a block in autophagy by increasing lysosomal pH. In this scenario, control myoblasts have fewer acidic vesicles and mimic the XMEA phenotype (Fig. 3B). In myotubes, however, the effect of chloroquine treatment in control cells is not observed. Nonetheless, both treated control myotubes and untreated and treated XMEA myotubes have fewer acidic vesicles compared to control myoblasts, suggesting that there is a further reduction in acidic vesicles with the pathology during differentiation (Fig. 3B).

### XMEA myotubes have increased autophagic flux

**Fig. 4.**
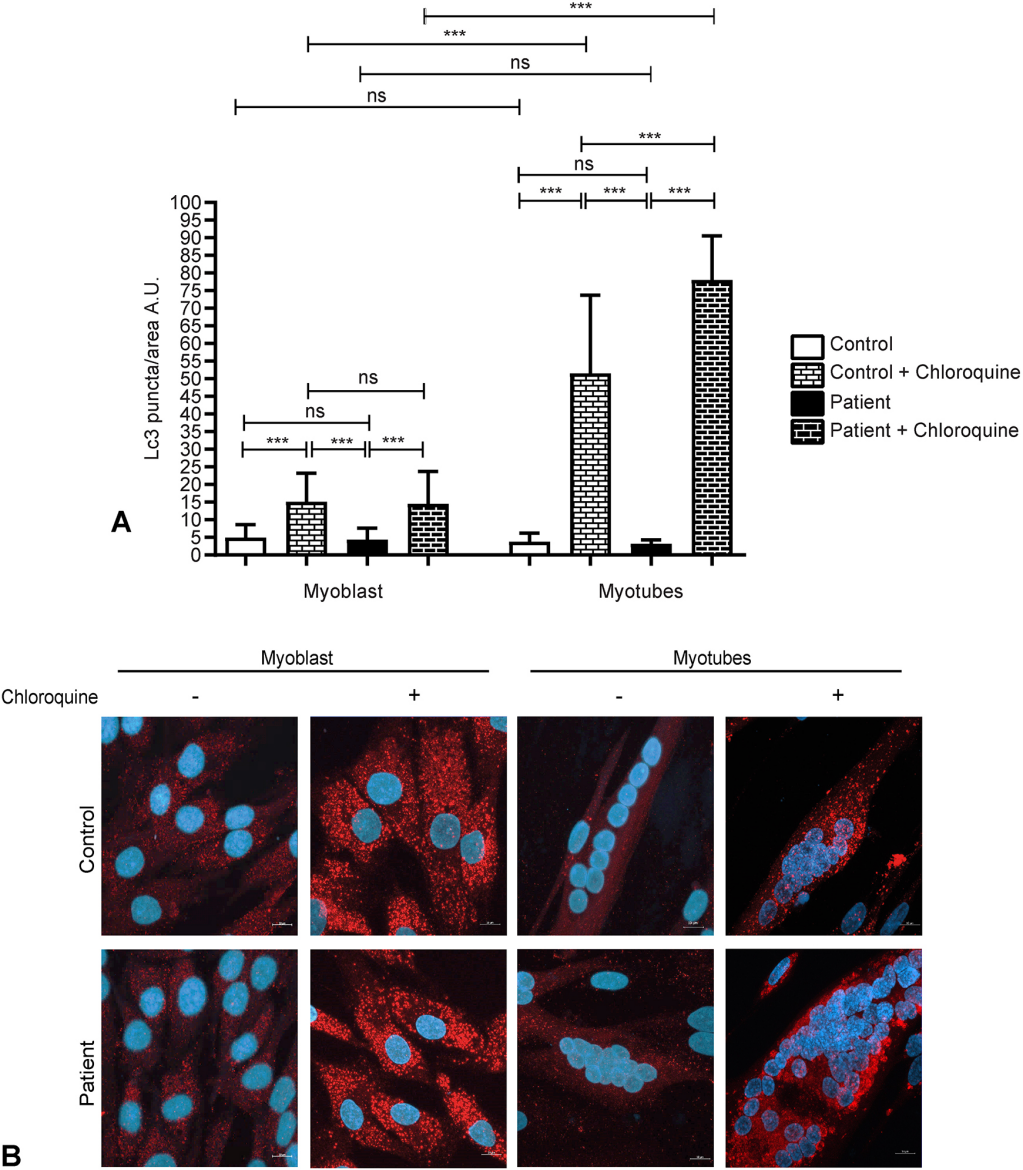
**Myotubes have increased autophagic flux compared to myoblasts, and patient myotubes have increased autophagic flux compared to control myotubes.** (A) Quantification of Lc3-positive vacuoles in control and patient myoblasts and myotubes with or without chloroquine treatment showed that myotubes accumulate more Lc3 vacuoles upon chloroquine treatment, indicating that they have increased autophagic flux. In addition, patient myotubes have more Lc3-positive vacuoles upon chloroquine treatment than control myotubes. (B) Representative images of control and patient myoblasts and myotubes immunostained for Lc3. Two independent experiments were performed, with 50 myoblasts or myotubes, with a sum of 50 nuclei evaluated per experiment. Red, Lc3; blue, DAPI. Magnification 630×, confocal microscopy. Scale bars: 10 µm. Data are presented as mean±s.d. ***P<0.0001; ns, non-significant. Three-way ANOVA with Bonferroni post hoc for multiple comparisons. A.U., arbitrary units.

To further investigate whether the expected alterations in autophagy with the disease were present in the initial steps of muscle formation, we investigated the presence of Lc3-positive vacuoles in control and patient-derived myoblasts and myotubes. No differences were observed between myoblasts and myotubes after 6 days of differentiation, and no distinctions could be observed between control and patient cells (Fig. 4). However, cells were cultured in serum-supplemented medium, thus autophagy is less active and Lc3-positive vacuole analysis might not reflect differences between control and patient-derived cells.

Considering that reduced levels of Lc3 can be related to either low autophagic activity or higher autophagic flux, we treated the cells with chloroquine, a known inhibitor of lysosomal degradation (Mizushima et al., 2010). Upon chloroquine treatment, there was an increase in the number of Lc3-positive vacuoles per area in myoblasts and myotubes, in control and patient-derived cells. Additionally, there was a higher Lc3-positive vacuole number per area in chloroquine-treated myotubes compared to myoblasts, demonstrating that myotubes have increased autophagic flux, which is more evident after the inhibition of lysosomal degradation (Fig. 4). Moreover, with chloroquine treatment, myotubes from patients have a higher number of Lc3-positive vacuoles per area, indicating that patient myotubes have increased autophagic flux compared to control myotubes.

**Fig. 5.**
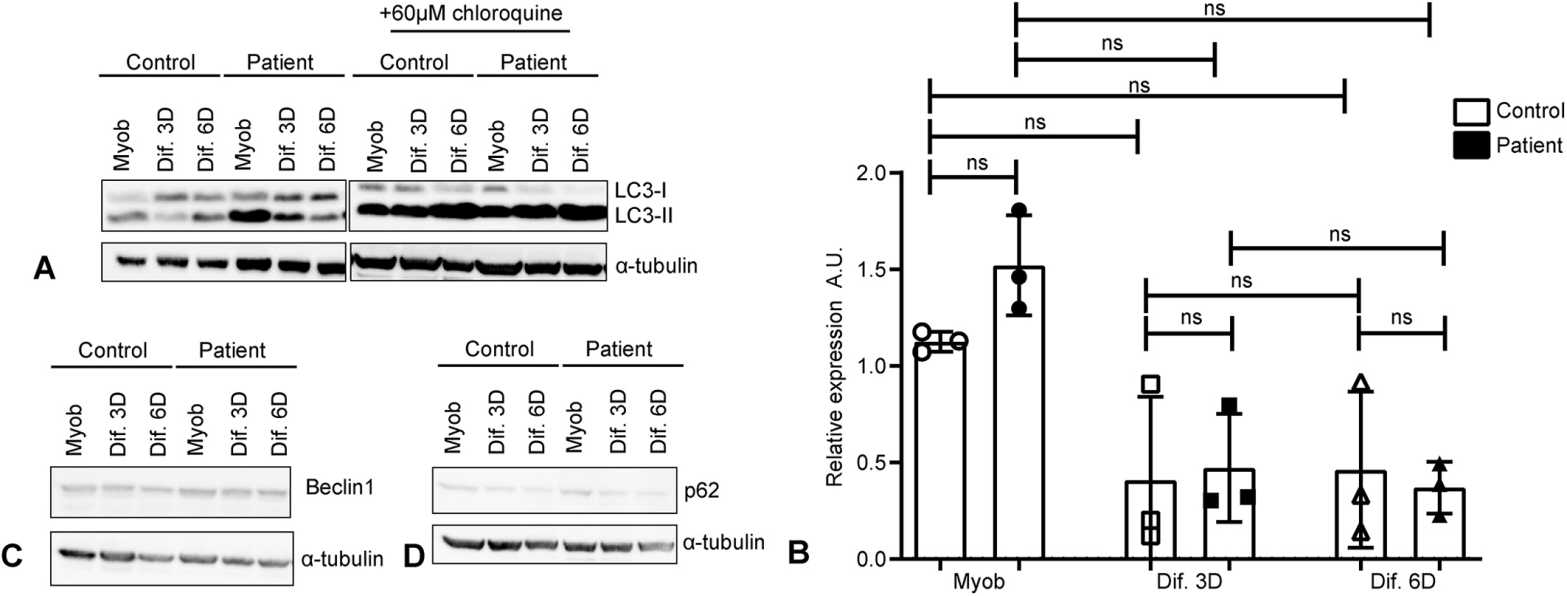
**Myotubes have increased autophagic flux observed by increased Lc3-II levels after chloroquine treatment.** XMEA-derived myotubes have increased levels of Lc3-II after chloroquine treatment and decreased p62 levels, indicating an increase in autophagic flux. (A) Representative images of Lc3 western blotting in cells with or without chloroquine treatment, showing that myoblasts have increased Lc3-II levels. Chloroquine treatment further increased Lc3-II in myotubes, but not myoblasts, indicating an increase in autophagy flux with differentiation. (B) Densitometric quantification showed a tendency for higher basal Lc3-II levels in myoblasts. Three independent experiments were performed. Individual points were plotted±s.d. ns, non-significant. One-way ANOVA (*P*=0.0327) with Dunn's post hoc for multiple comparisons. (C) Representative images of beclin 1 western blotting showing that its levels do not change with differentiation or XMEA pathology. Three independent experiments were performed. (D) Representative images of p62 western blotting showing that there is a slight decrease in p62 content in myotubes, especially XMEA-derived ones. Three independent experiments were performed. A.U., arbitrary units.

We next analyzed levels of Lc3-II, the membrane-bound form of Lc3 protein, by western blotting. Although not statistically significant, a trend for increased levels of this protein was observed in myoblasts compared to myotubes, in both control and patient-derived cells (Fig. 5A,B). In agreement with the immunofluorescence analysis, after chloroquine treatment, the levels of Lc3-II showed a tendency to increase in control and patient myotubes (Fig. 5A,B). This observation highlights that myotubes have increased autophagic flux, which is more evident after blockage of lysosomal degradation.

Additional studies of autophagy induction proteins, such as beclin1 and p62 (also known as SQSTM1), were performed. Beclin 1 analysis showed no differences between control and patient-derived cells (Fig. 5C). However, p62 levels slightly decreased with myotube formation (Fig. 5D), especially in patient-derived cells, which correlates with increased autophagic flux.

### Improved fusion of myoblasts in XMEA cells

**Fig. 6.**
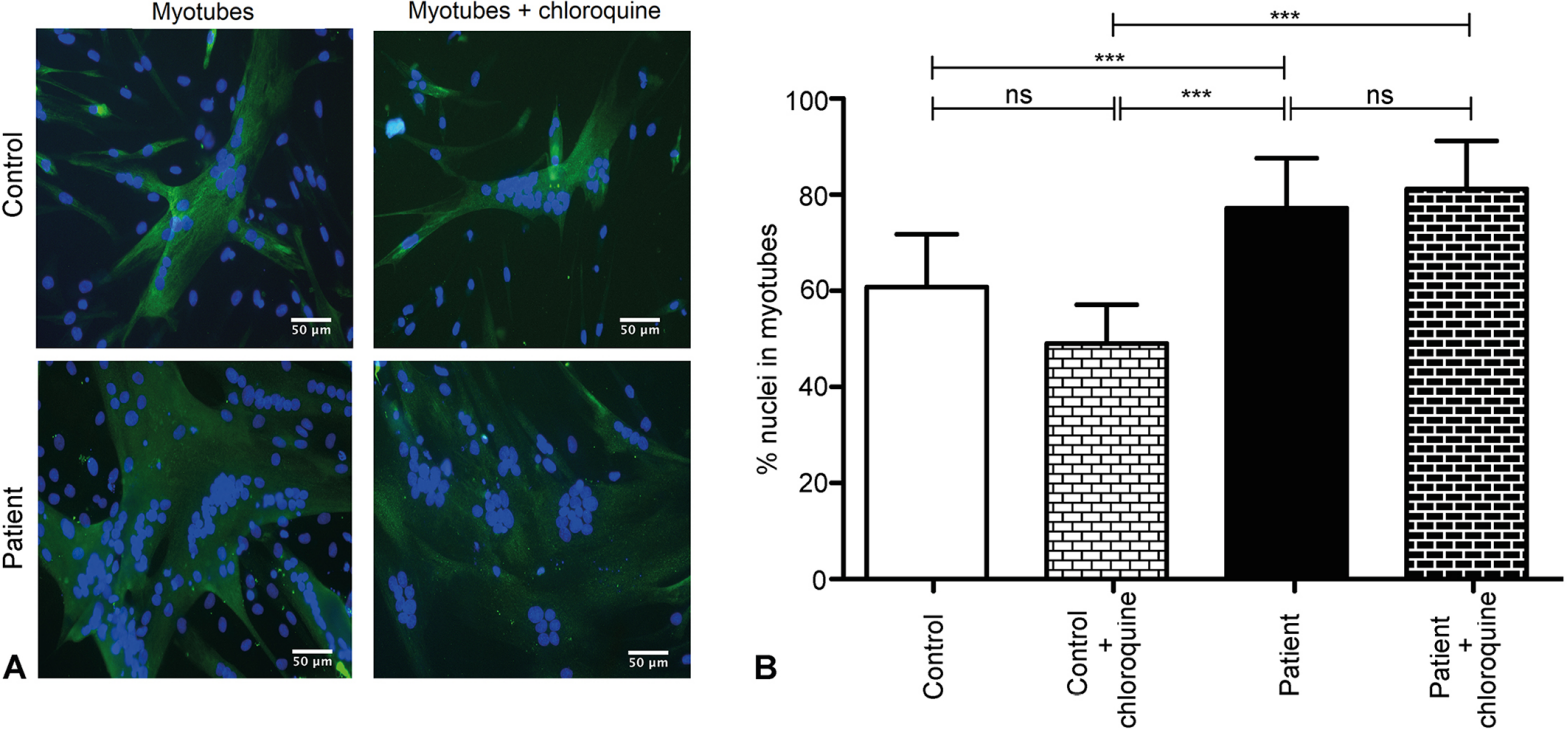
**Improved fusion of myoblasts in XMEA cells.** (A) Representative images of control and patient cells immunostained for myosin heavy chain after 3 days of myotube differentiation with or without chloroquine treatment. Scale bars: 50 μm. (B) Fusion index calculated in control and patient cells, with and without chloroquine treatment. The fusion index was higher in the patient-derived cells than in the control cells, and treatment with chloroquine did not affect the percentage of nuclei in myotubes. We measured 45 fields for control differentiated myotubes, 35 fields for patient myotubes, and five fields for chloroquine-treated control and patient myotubes differentiated for 3 days. Three independent experiments were performed for untreated cells and one for chloroquine-treated cells. Magnification 200×, confocal microscopy. Green, myosin heavy chain; blue, DAPI. Data are presented as mean±s.d. ***P<0.001; ns, non-significant. Two-way ANOVA with Bonferroni post hoc for multiple comparisons.

We next investigated whether differentiation was altered in XMEA cells, because visually a clear difference in myotube formation was observed, as in Fig. S1B. First, we evaluated the capacity of myoblasts to fuse and form myosin-heavy-chain-positive myotubes. We then calculated the fusion index, which corresponds to the percentage of nuclei that are inside differentiated cells. A total of 11,066 nuclei were counted in control cells, and 13,334 in patient-derived cells, after 3 days of differentiation. Interestingly, XMEA myotubes had a ∼10% higher fusion index compared to control myotubes, and this difference was statistically significant (Fig. 6A,B).

Additional experiments aimed to investigate whether the increase in myoblast fusion was due to the defective lysosomal acidification. Control and patient myotubes were differentiated for 3 days and treated with chloroquine. In the chloroquine-treated myotubes, 277 nuclei were counted for control cells and 324 nuclei for XMEA cells. We did not observe any differences in myotube formation following chloroquine treatment (Fig. 6A,B).

### Expression of myogenic genes and myofiber formation are not altered in XMEA cells

**Fig. 7.**
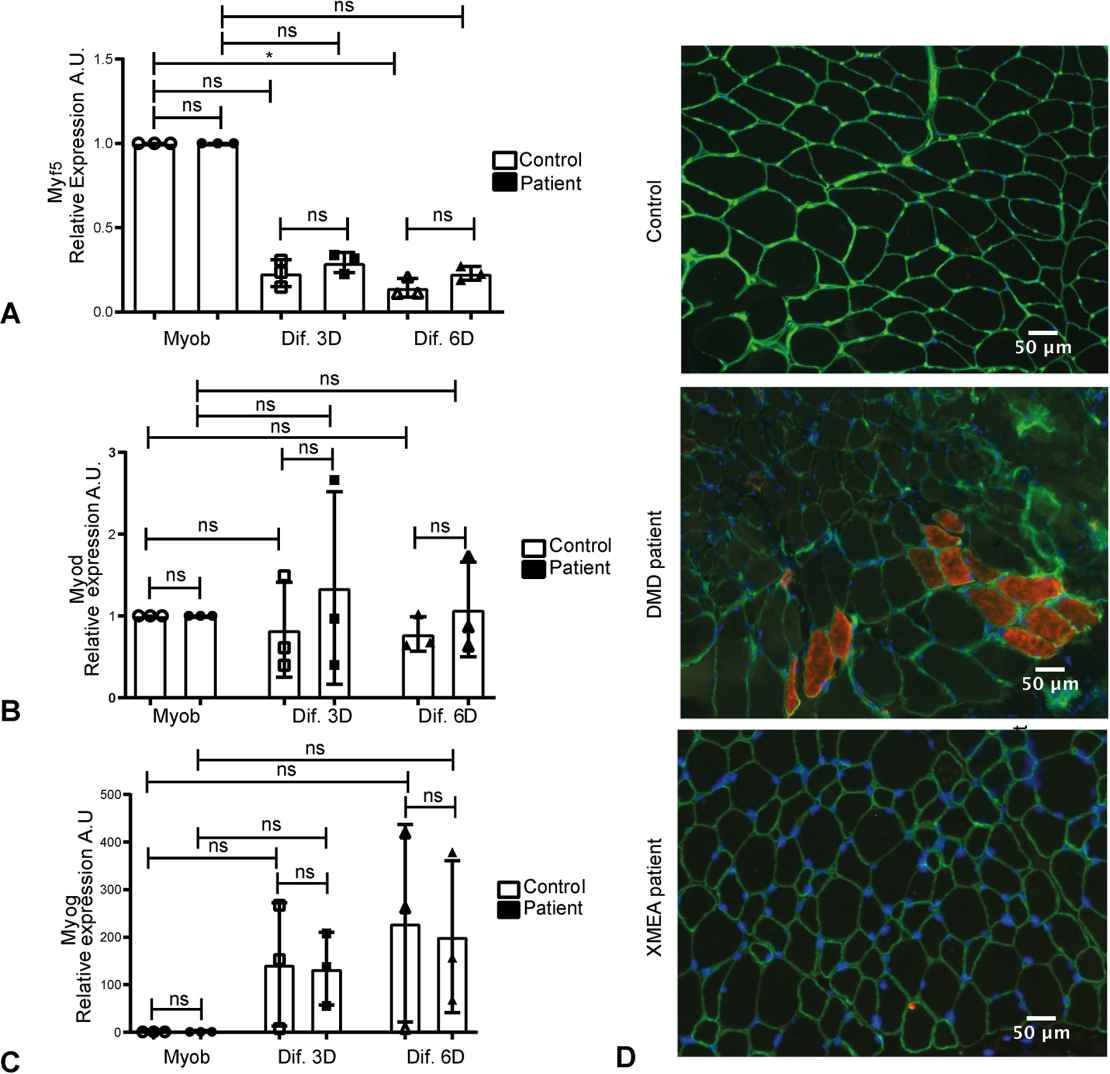
**The expression of genes involved in myogenesis is not altered in XMEA patient-derived cells.** (A) *MYF5* expression showed a trend to decrease in myotubes in control and patient cells. (B) *MYOD* expression decreased with myotube formation in control and patient cells, although the differences were not statistically significant. (C) *MYOG* expression increased with myotube formation in control and patient cells, although the differences were not statistically significant. Three independent experiments were performed. Individual points were plotted±s.d. *P<0.05; ns, non-significant. One-way ANOVA (*P*=0.0153 for *MYF5*, *P*=0.8798 for *MYOD* and *P*=0.0381for *MYOG*) with Dunn's post hoc for multiple comparisons. (D) Developmental myosin staining showing that the XMEA patient has no regenerating fibers, in contrast to a Duchenne Muscular Dystrophy (DMD) patient. Magnification 200×, fluorescent microscopy. Green, laminin; red, developmental myosin; blue, DAPI. Scale bars: 50 μm. A.U., arbitrary units.

We then examined the expression of genes involved in myogenesis, aiming to understand whether the higher fusion index observed in patient-derived cells could be due to alteration in their myogenic program. Our findings suggest that there are no alterations in the genes expressed in myoblasts, such as *MYOD* (also known as *MYOD1*) and *MYF5*, or in the expression of *MYOG*, a gene involved in differentiation, in myotubes (Fig. 7A-C).

Moreover, we evaluated whether the enhanced formation of myotubes in XMEA cells would result in the formation of new muscle fibers in the patient's muscle biopsy. After staining his muscle sections for development myosin heavy chain (dMHC), we did not observe new muscle fibers being formed in the XMEA biopsy, in comparison to a positive Duchenne muscular dystrophy (DMD) biopsy, in which regeneration and formation of new fibers is observed (Fig. 7D).

## DISCUSSION

In this study, we describe the first Brazilian family with a pathology suggestive of XMEA. The proband showed clinical symptoms and histological findings compatible with this diagnosis. Alongside molecular findings by next-generation sequencing (NGS), the patient included in this study fulfilled the histological and molecular criteria for XMEA. Muscle biopsy analysis with H&E staining showed variation in fiber size alongside basophilic inclusions, which are consistent with several case reports from XMEA patients (Chabrol et al., 2001; Villanova et al., 1995; Kalimo et al., 1988; Chow et al., 2006; Kurashige et al., 2013; Ruggieri et al., 2015; Villard et al., 2000; Crockett et al., 2014). All other histological and immunofluorescence analyses were also compatible with the disease. In the first described cases, the demonstration of the autophagic nature of the vacuoles was concluded based on increased activity of lysosomal enzymes (Kalimo et al., 1988). Later, colocalization of the vacuoles with Lc3 was demonstrated in a vacuolar disease, possibly XMEA (Yan et al., 2005). Our results confirm this finding, showing the presence of numerous Lc3-positive inclusions in the muscle biopsy of a patient with a confirmed mutation in the *VMA21* gene and reduction in *VMA21* mRNA expression, which is nowadays known to be causative of the disease. These observations highlight the autophagic nature of the vacuoles, together with the structural findings in electron microscopy analysis.

We next established a cellular model for *VMA21* gene mutation studies from the patient with a confirmed XMEA pathology. Considering that the *VMA21* gene product is involved in the correct assembly of the v-ATPase, deficiency in this protein complex could culminate in defective muscle formation. Alterations in the v-ATPases are widely reported in association with diseases; however, in most cases, specific subunits are affected and a total absence of v-ATPases has never been reported (Forgac, 2007). Additionally, v-ATPases have a wide range of functions beside lysosomal acidification, such as membrane trafficking and fusion (Merkulova et al., 2015). Autophagy has an important role in muscle development and is the pathway altered in XMEA; therefore, we investigated it in progenitor immortalized muscle cells, i.e. myoblasts and myotubes, from the XMEA patient. We confirmed that the capacity of proliferation and differentiation *in vitro* is preserved, even with reduction of *VMA21* gene expression and lower acidification of XMEA myoblasts and myotubes. Intriguingly, we observed a trend for increased VMA21 expression in control myotubes, suggesting a novel link between myogenesis and VMA21 expression.

Our aim was also to understand how the pathophysiology of XMEA might be affecting autophagy during the process of muscle differentiation, since this pathway has a well-known role in myotube formation (Sin et al., 2016; Fortini et al., 2016; McMillan and Quadrilatero, 2014). We found increased autophagic flux in myotubes through the analysis of Lc3 protein, both in control and patient-derived cells. It is important to note that myogenesis is a timely regulated process, and 6 days of differentiation might represent an advanced state of myotube formation. All previous studies regarding autophagic status in myotube formation were performed in mouse muscle cells (Sin et al., 2016; Fortini et al., 2016; McMillan and Quadrilatero, 2014), and the only study in humans was performed with differentiation from tonsil-derived mesenchymal stem cells (Park et al., 2017). Therefore, this is the first study in human muscle cells to suggest activation of the autophagy pathway during normal muscle differentiation.

Interestingly, all observations related to the basal activation of the autophagic pathway were similar in control and patient-derived cells, which indicates that, at this point of muscle formation, the higher levels of autophagy activation expected with the disease, as described by Ramachandran et al. (2013), were not yet detectable. However, when we treat cells with chloroquine, we observe that patient myotubes have higher autophagic flux compared to control myotubes, indicating that XMEA myotubes indeed have greater induction of the autophagic pathway. Therefore, muscle vacuolation and accumulation of vesicles of undigested content are probably starting in later stages of muscle formation. It is also important to consider that, in Ramachandran et al. (2013), the autophagic pathway was investigated in fibroblasts and lymphoblast cells, which might have a distinct response from that of muscle cells.

In XMEA cells, an increased capacity to fuse and form myotubes was found. Considering that myogenesis is a tightly regulated process, with factors such as Myf5 and Myod being expressed in myoblasts and Myog in myotubes (Almeida et al., 2016; Bentzinger et al., 2012; Parker et al., 2003), we further investigated whether alterations in their expression could explain the enhanced fusion in XMEA cells. Although we observed the expected changes in expression during differentiation, no differences were found between the control and patient-derived cells. In addition, the enhanced myotube formation was not translated into new myofibers being formed in the XMEA muscle, suggesting that this is a highly disease-dependent feature. However, it could contribute to changes in fiber caliber, resulting in the observed variation in fiber sizes in the muscle biopsy. These observations suggest that deregulation of myogenic factors and regeneration of muscle fibers might not be playing a role in the XMEA-increased myoblast fusion. In this sense, in a recent study analyzing a model of myoblasts with a decline in fusion capacity, the authors also observed no alteration in the expression of myogenic factors such as Myog, highlighting the possibility of morphological alterations without changes in the myogenic program (Park et al., 2016). Previous studies have also found enhanced fusion in myoblasts upon genetic modulation of factors such as insulin-like growth factor-1 (IGF-1) (Jacquemin et al., 2007), mitogen-activated protein kinase kinase kinase kinase 4 (Map4k4) (Wang et al., 2013), matrix metallopeptidase 7 (MMP-7) (Caron et al., 1999), creatine kinase b (CKB) (Simionescu-Bankston et al., 2015) or stabilin-2 (Stab-2) (Park et al., 2016), emphasizing the number of distinct cellular pathways that, when altered, may lead to enhanced myotube formation. Upon treating cells with chloroquine, we did not observe changes in myoblast fusion, indicating that lysosomal acidification is not the major player regulating this process. However, since v-ATPase is known to have acidification-independent functions, we hypothesize that the primary defect of XMEA can lead to an increase in myoblast fusion. In this regard, a v-ATPAse study in *Caenorhabditis elegans* demonstrated that it acts as a negative regulator of cell–cell fusion (Kontani et al., 2005). We therefore speculate that, in XMEA cells, the expected lower number of assembled v-ATPases might alleviate the repression of this mechanism, resulting in the higher myoblast fusion in the patient. Nevertheless, it remains to be elucidated whether this repression also occurs in all mammals and, moreover, whether this would be the primary cause of the enhanced cell fusion in XMEA muscle cells.

We also cannot rule out that the differentiation stimulus is indeed perceived by XMEA cells, and, as myoblast fusion is a tightly regulated process that requires proper autophagy, those cells fuse in an uncontrolled manner. Then, regardless of a higher differentiation rate, the patient-derived myotubes might not be as functional as normal muscle cells, compromising their physiology. It is also intriguing as to why a ubiquitous mechanism such as lysosomal acidification and autophagy would affect mainly the muscle in XMEA patients. In this regard, the results of the present study are bringing new evidence on impaired muscle myogenesis, with excessive myotube fusion, in the context of the XMEA phenotype.

## MATERIALS AND METHODS

### Patient

One family with five affected males was identified, linked through female carriers, with a typical X-linked pattern of inheritance. Further studies were performed with the proband in the Human Genome and Stem Cell Research Center (HUG-CELL) after obtaining a consent form from his parents.

### Muscle biopsy and histology

A muscle biopsy from the biceps was obtained from the 5-year-old patient, for diagnostic purposes. The muscle sample acquired was divided to obtain myoblasts in cell culture or immediately frozen in liquid nitrogen after removal and stored at −70°C. Histological sections were made from this frozen biopsy using a cryostat (Microm HM 505E). Routine histological and histochemical analyses including staining for H&E, modified Gomori trichrome, succinate dehydrogenase, NADH, alkaline phosphatase, Sudan Black, periodic acid–Schiff, Cox Congo Red and ATPase at pH 9.4 and pH 4.3 were performed. The stained slides were observed by light microscopy (Zeiss).

### Molecular analysis

DNA was isolated from blood by standard methodology. NGS was first performed using a customized NGS panel, with 95 genes involved in neuromuscular disorders, with Illumina's Nextera kits for library preparation and custom capture of these genes. Subsequently, whole-exome sequencing was carried out. Libraries were sequenced as 100-bp end runs (average insert size of 300 bases). Read alignment and variant calling was performed by Illumina CASAVA 1.9 pipeline software. An average of 120 gigabytes in aligned sequences was produced per sample, yielding a genome sampling depth of ∼40× and more than 95% of the UCSC hg19 reference genome sampled at 10 reads or more. NGS alignment and genotyping was also performed using Broad Institute's Best Practices (BWA 0.7; GATKv2.4; HaplotypeCaller); however, no further variants in the linked region were identified. As a downstream variant analysis, the resulting variant list for each sample was filtered using the 1000 Genomes variants (phase 1 release v3), the NHLBI Exome Sequencing Project (ESP6500) variants and the recently created Online Archive of Brazilian Mutations – AbraOM (http://www.abraom.ib.usp.br/).

### Cell culture

Myoblasts from the XMEA patient were isolated using explants from his muscle biopsy. Control myoblasts were isolated from the quadriceps of an unaffected individual and were generously provided by Dr Vincent Mouly (Institut de Myologie, Paris, France). All cells were immortalized at the Institut de Myologie by a previously established protocol (Mamchaoui et al., 2011). Importantly, the maintenance of the proliferative and differentiation capacities from the original cell lines was assessed and confirmed.

The immortalized cell lines were seeded at a density of 2.5×10^5^ cells per 75 cm^2^ and grown in proliferation medium, Skeletal Muscle Cell Growth Medium (PromoCell), with 20% fetal bovine serum (Gibco). When cells reached 100% confluence, they were switched to differentiation medium, which is composed of DMEM GlutaMAX™ (Invitrogen), 50 μg/ml gentamycin (Invitrogen) and 10 μg/ml insulin (Sigma-Aldrich).

Proliferating and 3- and 6-day-differentiated cells were collected after incubation with trypsin (Gibco) and centrifuged at 300 ***g*** for 10 min at 4°C. The cell pellets were washed with 1× PBS (Gibco) and centrifuged once more. The resulting pellets were frozen and stored at −70°C before proceeding to RNA and protein analysis. Cells were obtained from three independent experiments.

### Immunofluorescence

Frozen muscle biopsy sections from the XMEA patient and controls were fixed with 100% methanol for 15 min at −20°C, permeabilized with 0.1% Triton X-100 (Sigma-Aldrich) for 5 min and blocked for 1 h in a solution of 1× PBS (Gibco), 5% goat serum (Sigma-Aldrich) and 0.3% Triton X-100 (Sigma-Aldrich). The histological sections were then incubated overnight at 4°C with primary antibody against Lc3 (3868, Cell Signaling Technology; 1:200) or dMHC (VP-M664, Vector Laboratories; 1:30) in a solution of 1× PBS (Gibco), 1% bovine serum albumin (Sigma-Aldrich) and 0.3% Triton X-100 (Sigma-Aldrich). Sections were washed three times in 1× PBS (Gibco), incubated with an anti-rabbit secondary antibody conjugated to Alexa Fluor 594 (11012, Invitrogen) for 1 h at room temperature and washed again. Sections were mounted with Vectashield^®^ Mounting Medium (Vector Laboratories) with 4′,6-diamidino-2-phenylindole (DAPI). The specificity of antibodies was assessed by using control samples and incubating samples with secondary antibody only. Images were obtained in a Zeiss LSM-800 confocal system with a Plan-Apochromat 63×/1.4 NA oil objective or a Zeiss microscope with epi-fluorescence.

Patient and control myoblasts were plated at 10^5^ cells per well in 6-well plates on glass coverslips. Cells were grown in proliferation medium until 70-80% confluence, when they were fixed and processed as above for Lc3 immunostaining. Another set of plates was allowed to reach 100% confluence, when they were switched to differentiation medium for 6 days. Afterwards, cells were also fixed and processed as described for muscle biopsy sections. The images obtained from two independent experiments were analyzed with ImageJ software to determine the intensity of Lc3-positive puncta per area.

### Fusion index

Myoblasts from the XMEA patient and control were plated at 10^5^ cells per well in 6-well plates on glass coverslips. Cells were grown in proliferation medium until they reached 100% confluence, when they were switched to differentiation medium for 3 or 6 days. Myotubes were fixed with 2% paraformaldehyde for 20 min, followed by permeabilization with 1× TBS with 0.1% Triton X-100 (Sigma-Aldrich) for 30 min and blocking in 1× TBS with 1% fetal bovine serum (Gibco) and 0.1% Triton X-100 (Sigma-Aldrich) for 30 min at room temperature. Cells were then incubated with an antibody against myosin heavy chain (MF-20, Developmental Studies Hybridoma Bank; 1:100) overnight at 4°C, followed by three washes with 1× TBS with 0.1% Triton X-100 (Sigma-Aldrich) and incubation with a secondary mouse antibody conjugated to Alexa Fluor 488 (11001, Invitrogen) and washed again. Slides were mounted with Vectashield^®^ Mounting Medium (Vector Laboratories) with DAPI. The specificity of antibodies was assessed by using undifferentiated control samples and incubating samples with secondary antibody only. Images were obtained in a Zeiss LSM-800 confocal system with an EC-Plan Neofluar 20×/0.5.

The fusion index was calculated as the number of nuclei inside myotubes/total number of nuclei, as measured from five fields at 20× magnification. Three independent experiments were quantified for patient-derived and control myotubes, at 3 and 6 days after switch to differentiation medium.

### Lysosensor analysis

Autophagic flux was evaluated based on the protocol described by Maulucci et al. (2015). Myoblasts were kept for 1 h in serum-free medium to be in the same condition as myotubes at the time of Lysosensor analysis. Myotubes were evaluated after 3 days of differentiation. Cells were incubated with 2 µM Lysosensor Yellow/Blue fluorescent probe (Invitrogen) at 37°C for 1 h. *Z*-stack images of single cells and myotubes were acquired in a Zeiss LSM-800 confocal system with an EC-Plan Neofluar 40×/0.5. The excitation filters used to obtain the images were 425 nm (emission of 446 nm, to see the less-acid vesicles, represented in blue) and 519 nm (emission of 543 nm, to see the more-acid vesicles, represented in yellow). Fluorescence signal was quantified on the sum projection of *Z*-stacks. Cells were manually outlined and the integrated density was measured using ImageJ software (https://imagej.nih.gov/ij/). Three random areas around the cells were measured to subtract background noise. Then, the ratio between yellow and blue fluorescence was calculated.

### Chloroquine treatment

Control and patient myoblasts and myotubes were incubated with 60 µM chloroquine (Abcam) diluted in proliferation or differentiation medium for 24 h. Cells were collected as described above and the cell pellet was processed for protein extraction. Patient and control myoblasts and myotubes were also cultivated in glass coverslips, and incubated with 60 µM chloroquine (Abcam) diluted in proliferation or differentiation medium for 24 h. Live cells were used for Lysosensor experiments. In addition, cells were fixed and processed as described above for Lc3 immunofluorescence. Finally, myotubes were fixed and processed for fusion index calculation.

### Flow cytometry analysis

Control and patient myoblasts were seeded at a density of 2.5×10^5^ cells per 75 cm^2^ and grown in proliferation medium until they reached 70-80% confluence. Cells were detached using trypsin (Gibco) and 10^6^ cells from each line were stained by incubating cells at 37°C with 250 nM MitoTracker Red FM (MM22425, Invitrogen) or 50 nM LysoTracker Red DND-99 (LL7528, Invitrogen), according to the manufacturer's instructions. Cells were then analyzed by a flow cytometer Aria (BD Biosciences) in three independent experiments. For mean fluorescence intensity determination, FlowJo software (https://www.flowjo.com/) was used.

### RNA analysis

Fragments of frozen muscle from the patient and controls and the previously obtained cell pellets were used for total RNA extraction using the TRIzol protocol (Invitrogen). The obtained RNA was solubilized in RNase-free water (Ambion™) and quantified in a Nanodrop spectrophotometer (Thermo Fisher Scientific). cDNA synthesis was performed using 1 µg total RNA according to the M-MLV protocol (Invitrogen). Specific sets of primers were used for analyses of autophagic and myogenic genes and *TBP* was used as an endogenous control (Table 1). Real-time PCR was performed in triplicate for *VMA21* gene expression analysis and duplicates for the remaining genes using the FastStart Universal SYBR Green Master Rox (Roche) on a thermocycler 7500 Fast (Applied Biosystems). The fold change was obtained by the 2^−ΔΔCT^ method, with *TBP* and the control muscles or myoblasts as normalizers for autophagic genes, and the respective undifferentiated samples for myogenic genes.

**Table 1.**
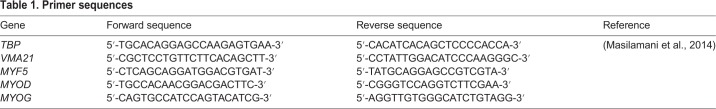
**Primer sequences**

### Protein analysis

Cell pellets were homogenized in RIPA buffer (Sigma-Aldrich) supplemented with 1% protease and phosphatase inhibitors (Sigma-Aldrich). Samples were kept on ice for 30 min and afterwards centrifuged at 16,000 ***g*** for 20 min at 4°C, followed by protein quantification by the bicinchoninic acid assay (Pierce, Thermo Fisher Scientific). Homogenized samples were separated by SDS-PAGE, and transferred to nitrocellulose membranes (Amersham Pharmacia). The membranes were then probed using the following antibodies: anti-Lc3 (3868, Cell Signaling Technology; 1:1000), anti-p62 (5114, Cell Signaling Technology; 1:200), anti-beclin1 (sc-11427, Santa Cruz Biotechnology; 1:500) and anti-α-tubulin (T5168, Sigma-Aldrich; 1:1000). The specificity of antibodies was assessed using positive controls. The membranes were then incubated with the appropriate secondary horseradish peroxidase-conjugated anti-rabbit or anti-mouse antibodies (Thermo Fisher Scientific) and revealed using a Novex™ ECL Chemiluminescent Substrate Reagent kit (Invitrogen). For the reaction with the endogenous control, membranes were stripped with a stripping buffer (Thermo Fisher Scientific) and re-probed for α-tubulin analysis. Bands were quantified by densitometry using ImageJ.

### Statistical analysis

Data were expressed as mean±s.d. Differences between each control and the patient and between myoblasts and myotubes were assessed by one-way ANOVA with Dunn's post hoc for multiple comparisons in cases of quantitative PCR or western blotting experiments that were performed three independent times. Two-way ANOVA with Bonferroni post hoc for multiple comparisons was used when analyzing data obtained for fusion index experiments, comparing myoblasts and myotubes, untreated or treated with chloroquine. For Lysosensor and Lc3 vacuole detection experiments comparing myoblasts and myotubes, treatments and controls and the patient, three-way ANOVA with Bonferroni post hoc for multiple comparisons was used. All analyses were performed using GraphPad Prism. *P*<0.05 was considered significant.

This article is part of a special collection ‘A Guide to Using Neuromuscular Disease Models for Basic and Preclinical Studies’, which was launched in a dedicated issue guest edited by Annemieke Aartsma-Rus, Maaike van Putten and James Dowling. See related articles in this collection at http://dmm.biologists.org/collection/neuromuscular.

## Supplementary Material

Supplementary information

## References

[DMM041244C1] Almeida C. F., Fernandes S. A., Ribeiro Junior A. F., Keith Okamoto O. and Vainzof M. (2016). Muscle satellite cells: exploring the basic biology to rule them. *Stem Cells Int.* 2016, 1078686 10.1155/2016/1078686 27042182 PMC4794588

[DMM041244C2] Bentzinger C. F., Wang Y. X. and Rudnicki M. A. (2012). Building muscle: molecular regulation of myogenesis. *Cold Spring Harb. Perspect. Biol.* 4, a008342 10.1101/cshperspect.a008342 22300977 PMC3281568

[DMM041244C3] Caron N. J., Asselin I., Morel G. and Tremblay J. P. (1999). Increased myogenic potential and fusion of matrilysin-expressing myoblasts transplanted in mice. *Cell Transplant.* 8, 465-476. 10.1177/096368979900800502 10580341

[DMM041244C4] Chabrol B., Figarella-Branger D., Coquet M., Mancini J., Fontan D., Pedespan J. M., Francannet C., Pouget J., Beaufrère A. M. and Pellissier J. F. (2001). X-linked myopathy with excessive autophagy: a clinicopathological study of five new families. *Neuromuscul. Disord.* 11, 376-388. 10.1016/S0960-8966(00)00209-1 11369189

[DMM041244C5] Chow G., Beesley C. E., Robson K., Winchester B. G. and Holton J. L. (2006). Case of X-linked myopathy with excessive autophagy. *J. Child Neurol.* 21, 431-433. 16901453

[DMM041244C6] Crockett C. D., Ruggieri A., Gujrati M., Zallek C. M., Ramachandran N., Minassian B. A. and Moore S. A. (2014). Late adult-onset of X-linked myopathy with excessive autophagy. *Muscle Nerve* 50, 138-144. 10.1002/mus.24197 24488655 PMC4589296

[DMM041244C7] Forgac M. (2007). Vacuolar ATPases: rotary proton pumps in physiology and pathophysiology. *Nat. Rev. Mol. Cell Biol.* 8, 917-929. 10.1038/nrm2272 17912264

[DMM041244C8] Fortini P., Ferretti C., Iorio E., Cagnin M., Garribba L., Pietraforte D., Falchi M., Pascucci B., Baccarini S., Morani F. et al. (2016). The fine tuning of metabolism, autophagy and differentiation during in vitro myogenesis. *Cell Death Dis.* 7, e2168 10.1038/cddis.2016.50 27031965 PMC4823951

[DMM041244C9] Jacquemin V., Butler-Browne G. S., Furling D. and Mouly V. (2007). IL-13 mediates the recruitment of reserve cells for fusion during IGF-1-induced hypertrophy of human myotubes. *J. Cell Sci.* 120, 670-681. 10.1242/jcs.03371 17264150

[DMM041244C10] Kalimo H., Savontaus M.-L., Lang H., Paljärvi L., Sonninen V., Dean P. B., Katevuo K. and Salminen A. (1988). X-linked myopathy with excessive autophagy: a new hereditary muscle disease. *Ann. Neurol.* 23, 258-265. 10.1002/ana.410230308 2897824

[DMM041244C11] Kontani K., Moskowitz I. P. G. and Rothman J. H. (2005). Repression of cell-cell fusion by components of the C. elegans vacuolar ATPase complex. *Dev. Cell* 8, 787-794. 10.1016/j.devcel.2005.02.018 15866168

[DMM041244C12] Kurashige T., Takahashi T., Yamazaki Y., Nagano Y., Kondo K., Nakamura T., Yamawaki T., Tsuburaya R., Hayashi Y. K., Nonaka I. et al. (2013). Elevated urinary β2 microglobulin in the first identified Japanese family afflicted by X-linked myopathy with excessive autophagy. *Neuromuscul. Disord.* 23, 911-916. 10.1016/j.nmd.2013.06.003 23850239

[DMM041244C13] Mamchaoui K., Trollet C., Bigot A., Negroni E., Chaouch S., Wolff A., Kandalla P. K., Marie S., Di Santo J., St Guily J. L. et al. (2011). Immortalized pathological human myoblasts: towards a universal tool for the study of neuromuscular disorders. *Skelet. Muscle* 1, 34 10.1186/2044-5040-1-34 22040608 PMC3235972

[DMM041244C14] Mammucari C., Milan G., Romanello V., Masiero E., Rudolf R., Del Piccolo P., Burden S. J., Di Lisi R., Sandri C., Zhao J. et al. (2007). FoxO3 controls autophagy in skeletal muscle in vivo. *Cell Metab.* 6, 458-471. 10.1016/j.cmet.2007.11.001 18054315

[DMM041244C15] Masiero E., Agatea L., Mammucari C., Blaauw B., Loro E., Komatsu M., Metzger D., Reggiani C., Schiaffino S. and Sandri M. (2009). Autophagy is required to maintain muscle mass. *Cell Metab.* 10, 507-515. 10.1016/j.cmet.2009.10.008 19945408

[DMM041244C16] Masilamani T. J., Loiselle J. J. and Sutherland L. C. (2014). Assessment of reference genes for real-time quantitative PCR gene expression normalization during C2C12 and H9c2 skeletal muscle differentiation. *Mol. Biotechnol.* 56, 329-339. 10.1007/s12033-013-9712-2 24146429

[DMM041244C17] Maulucci G., Chiarpotto M., Papi M., Samengo D., Pani G. and De Spirito M. (2015). Quantitative analysis of autophagic flux by confocal pH-imaging of autophagic intermediates. *Autophagy* 11, 1905-1916. 10.1080/15548627.2015.1084455 26506895 PMC4824579

[DMM041244C18] Mcmillan E. M. and Quadrilatero J. (2014). Autophagy is required and protects against apoptosis during myoblast differentiation. *Biochem. J.* 462, 267-277. 10.1042/BJ20140312 24865278

[DMM041244C19] Merkulova M., Păunescu T. G., Azroyan A., Marshansky V., Breton S. and Brown D. (2015). Mapping the H^+^ (V)-ATPase interactome: identification of proteins involved in trafficking, folding, assembly and phosphorylation. *Sci. Rep.* 5, 14827 10.1038/srep14827 26442671 PMC4595830

[DMM041244C20] Minassian B. A., Aiyar R., Alic S., Banwell B., Villanova M., Fardeau M., Mandell J. W., Juel V. C., Rafii M., Auranen M. et al. (2002). Narrowing in on the causative defect of an intriguing X-linked myopathy with excessive autophagy. *Neurology* 59, 596-601. 10.1212/WNL.59.4.596 12196656

[DMM041244C21] Mizushima N., Yoshimori T. and Levine B. (2010). Methods in mammalian autophagy research. *Cell* 140, 313-326. 10.1016/j.cell.2010.01.028 20144757 PMC2852113

[DMM041244C22] Munteanu I., Kalimo H., Saraste A., Nishino I. and Minassian B. A. (2017). Cardiac autophagic vacuolation in severe X-linked myopathy with excessive autophagy. *Neuromuscul. Disord.* 27, 185-187. 10.1016/j.nmd.2016.10.007 27916343

[DMM041244C23] Park S. Y., Yun Y., Lim J. S., Kim M. J., Kim S. Y., Kim J. E. and Kim I. S. (2016). Stabilin-2 modulates the efficiency of myoblast fusion during myogenic differentiation and muscle regeneration. *Nat. Commun.* 7, 10871 10.1038/ncomms10871 26972991 PMC4793076

[DMM041244C24] Park S., Choi Y., Jung N., Kim J., Oh S., Yu Y., Ahn J.-H., Jo I., Choi B.-O. and Jung S.-C. (2017). Autophagy induction in the skeletal myogenic differentiation of human tonsil-derived mesenchymal stem cells. *Int. J. Mol. Med.* 39, 831-840. 10.3892/ijmm.2017.2898 28259927 PMC5360438

[DMM041244C25] Parker M. H., Seale P. and Rudnicki M. A. (2003). Looking back to the embryo: defining transcriptional networks in adult myogenesis. *Nat. Rev. Genet.* 4, 497-507. 10.1038/nrg1109 12838342

[DMM041244C26] Ramachandran N., Munteanu I., Wang P., Ruggieri A., Rilstone J. J., Israelian N., Naranian T., Paroutis P., Guo R., Ren Z.-P. et al. (2013). VMA21 deficiency prevents vacuolar ATPase assembly and causes autophagic vacuolar myopathy. *Acta Neuropathol.* 125, 439-457. 10.1007/s00401-012-1073-6 23315026

[DMM041244C27] Ruggieri A., Ramachandran N., Wang P., Haan E., Kneebone C., Manavis J., Morandi L., Moroni I., Blumbergs P., Mora M. et al. (2015). Non-coding VMA21 deletions cause X-linked myopathy with excessive autophagy. *Neuromuscul. Disord.* 25, 207-211. 10.1016/j.nmd.2014.11.014 25683699

[DMM041244C28] Saraste A., Koskenvuo J. W., Airaksinen J., Ramachandran N., Munteanu I., Udd B., Huovinen S., Kalimo H. and Minassian B. A. (2015). No cardiomyopathy in X-linked myopathy with excessive autophagy. *Neuromuscul. Disord.* 25, 485-487. 10.1016/j.nmd.2015.03.003 25845477

[DMM041244C29] Saviranta P., Lindlöf M., Lehesjoki A. E., Kalimo H., Lang H., Sonninen V., Savontaus M. L. and De La Chapelle A. (1988). Linkage studies in a new X-linked myopathy, suggesting exclusion of DMD locus and tentative assignment to distal Xq. *Am. J. Hum. Genet.* 42, 84-88. 2892402 PMC1715330

[DMM041244C30] Simionescu-Bankston A., Pichavant C., Canner J. P., Apponi L. H., Wang Y., Steeds C., Olthoff J. T., Belanto J. J., Ervasti J. M. and Pavlath G. K. (2015). Creatine kinase B is necessary to limit myoblast fusion during myogenesis. *Am. J. Physiol. Cell Physiol.* 308, C919-C931. 10.1152/ajpcell.00029.2015 25810257 PMC4451350

[DMM041244C31] Sin J., Andres A. M., Taylor D. J. R., Weston T., Hiraumi Y., Stotland A., Kim B. J., Huang C., Doran K. S. and Gottlieb R. A. (2016). Mitophagy is required for mitochondrial biogenesis and myogenic differentiation of C2C12 myoblasts. *Autophagy* 12, 369-380. 10.1080/15548627.2015.1115172 26566717 PMC4836019

[DMM041244C32] Villanova M., Louboutin J. P., Chateau D., Eymard B., Sagniez M., Toméa F. M. S. and Fardeau M. (1995). X-linked vacuolated myopathy: complement membrane attack complex on surface membrane of injured muscle fibers. *Ann. Neurol.* 37, 637-645. 10.1002/ana.410370514 7755359

[DMM041244C33] Villard L., Des Portes V., Levy N., Louboutin J.-P., Recan D., Coquet M., Chabrol B., Figarella-Branger D., Chelly J., Pellissier J.-F. et al. (2000). Linkage of X-linked myopathy with excessive autophagy (XMEA) to Xq28. *Eur. J. Hum. Genet.* 8, 125-129. 10.1038/sj.ejhg.5200432 10757644

[DMM041244C34] Wang M., Amano S. U., Flach R. J. R., Chawla A., Aouadi M. and Czech M. P. (2013). Identification of Map4k4 as a novel suppressor of skeletal muscle differentiation. *Mol. Cell. Biol.* 33, 678-687. 10.1128/MCB.00618-12 23207904 PMC3571342

[DMM041244C35] Yan C., Tanaka M., Sugie K., Nobutoki T., Woo M., Murase N., Higuchi Y., Noguchi S., Nonaka I., Hayashi Y. K. et al. (2005). A new congenital form of X-linked autophagic vacuolar myopathy. *Neurology* 65, 1132-1134. 10.1212/01.wnl.0000178979.19887.f5 16217076

[DMM041244C36] Zhao J., Brault J. J., Schild A., Cao P., Sandri M., Schiaffino S., Lecker S. H. and Goldberg A. L. (2007). FoxO3 coordinately activates protein degradation by the autophagic/lysosomal and proteasomal pathways in atrophying muscle cells. *Cell Metab.* 6, 472-483. 10.1016/j.cmet.2007.11.004 18054316

