# Supplementary material for: Altered in vitro muscle differentiation in X-linked myopathy with excessive autophagy
Source: Dis Model Mech. 2020 Jan 10;13(2):dmm041244. doi: 10.1242/dmm.041244 (PMC6994946; doi:10.1242/dmm.041244)
Supplement: Supplementary information [file dmm-13-041244-s1.pdf]

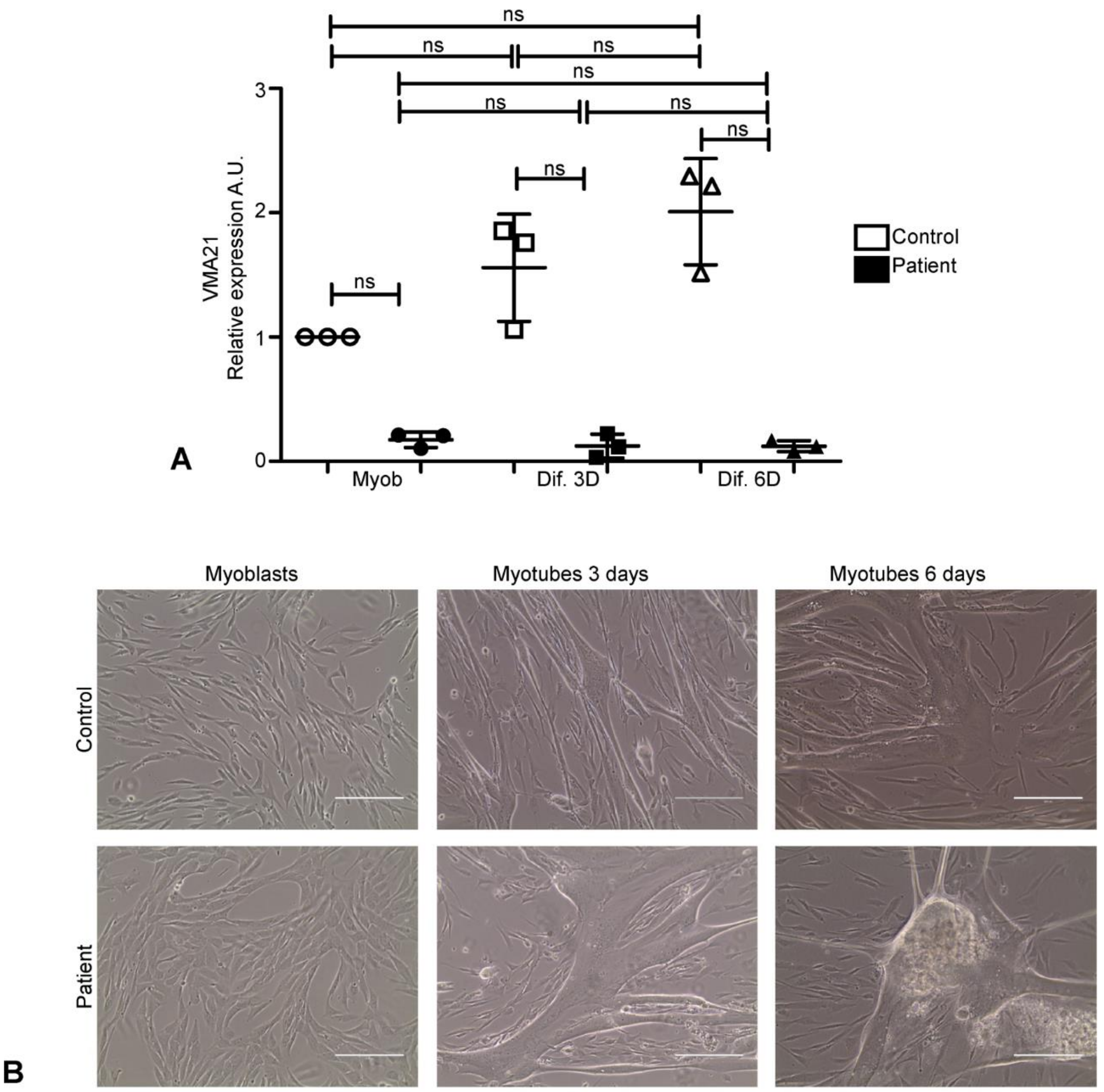

Figure S1. Patient and control progenitor muscle cells were successfully obtained and immortalized. (A) Reduced expression of *VMA21* gene expression was observed in patient-derived myoblasts and myotubes, although not statistically significant. Three independent experiments were performed. Individual points were plotted± SD. ns non-significant, One-way ANOVA test ($p = 0.0126$) with Dunn's post hoc for multiple comparisons. (B) Representative images of control and patient myoblasts and myotubes differentiated for three or six days. Magnification 200x, phase contrast microscopy.

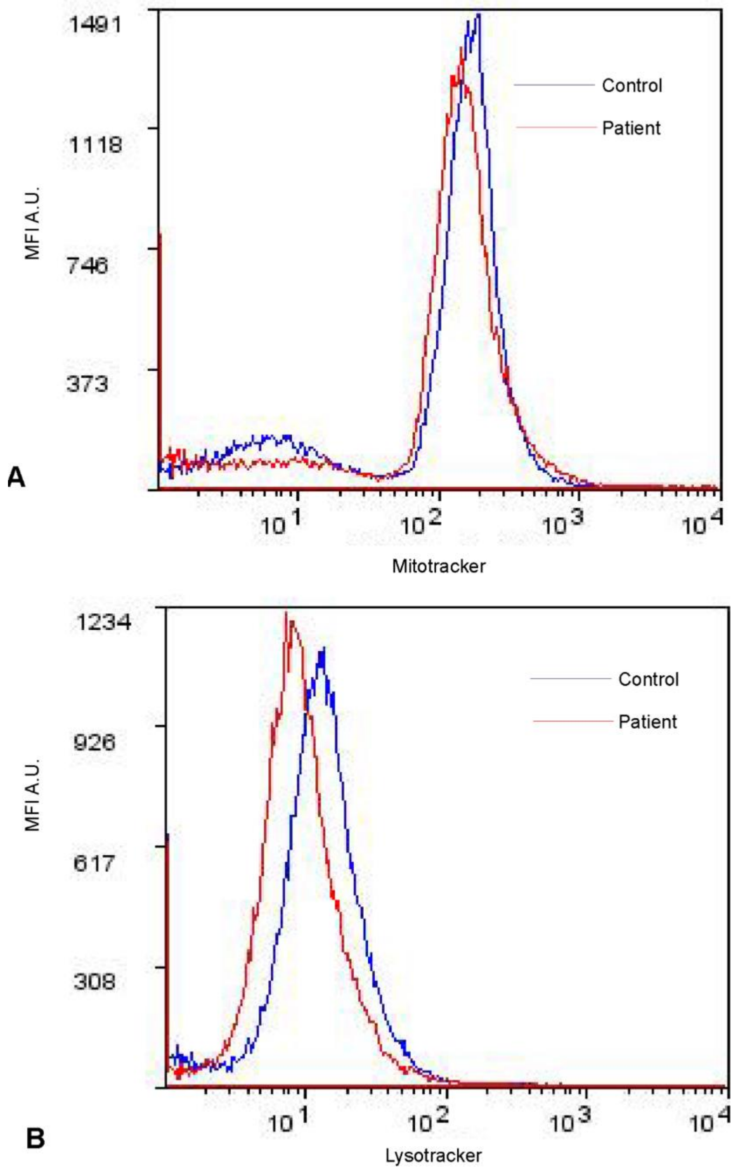

Figure S2. Patient myoblasts have less lysosomal staining with the Lysotracker probe. (A) Representative plot of median fluorescence intensity of Mitotracker shows no differences in mitochondrial content. (B) Representative plot of median fluorescence intensity of Lysotracker shows that patient cells have lower staining with this probe. Three independent experiments were performed.
